# Profiling the Effects of Repetitive Morphine Administration on Motor Behavior in Rats

**DOI:** 10.3390/molecules26144355

**Published:** 2021-07-19

**Authors:** Alok K. Paul, Nuri Gueven, Nikolas Dietis

**Affiliations:** 1 School of Pharmacy and Pharmacology, University of Tasmania, Hobart, TAS 7001, Australia; nuri.guven@utas.edu.au; 2 Medical School, University of Cyprus, Nicosia 1678, Cyprus

**Keywords:** morphine dosing, behavior, locomotor activity, tolerance

## Abstract

Efficient repetitive clinical use of morphine is limited by its numerous side effects, whereas analgesic tolerance necessitates subsequent increases in morphine dose to achieve adequate levels of analgesia. While many studies focused on analgesic tolerance, the effect of morphine dosing on non-analgesic effects has been overlooked. This study aimed to characterize morphine-induced behavior and the development and progression of morphine-induced behavioral tolerance. Adult male Sprague–Dawley rats were repetitively treated with subcutaneous morphine for 14 days in two dose groups (A: 5 mg/kg/day (b.i.d.) → 10 mg/kg/day; B: 10 mg/kg/day (b.i.d.) → 20 mg/kg/day). Motor behavior was assessed daily (distance traveled, speed, moving time, rearing, rotation) in an open-field arena, before and 30 min post-injections. Antinociception was measured using tail-flick and hot-plate assays. All measured parameters were highly suppressed in both dosing groups on the first treatment day, followed by a gradual manifestation of behavioral tolerance as the treatment progressed. Animals in the high-dose group showed increased locomotor activity after 10 days of morphine treatment. This excitatory phase converted to an inhibition of behavior when a higher morphine dose was introduced. We suggest that the excitatory locomotor effects of repetitive high-dose morphine exposure represent a signature of its behavioral and antinociceptive tolerance.

## 1. Introduction

Long-term clinical use of opioids such as morphine is limited by its significant side effects such as drowsiness, itching, respiratory depression, constipation, addiction, and dependence [1,2,3]. Although predicting the appearance of morphine-induced side effects is important for effective pain relief, the relationship between opioid dosing and the appearance of drug-induced side effects is currently not well established. In the clinic, pain relief and side effects appear to correlate poorly [4]. Behavioral side effects of morphine in different clinical studies are described as dose-dependent, such as pruritus [5], and dose-independent, such as nausea and vomiting [6,7]. It therefore is important to understand how the dosing regimen can affect behavioral effects. Noticeably, antinociceptive tolerance largely depends on morphine dose and dosing protocol, and a high starting dose or a high follow-up dose of morphine produces less antinociceptive tolerance [8]. We previously measured antinociception and antinociceptive tolerance in rats using four different morphine dosing regimens. Antinociception and antinociceptive tolerance were measured using two independent assays (tail-flick and hot-plate assays) [8]. Behavioral measurements are more complex than nociceptive pain measurements, and therefore, only two morphine dosing regimens were selected for the present study. Our previous study confirmed the manifestation of antinociceptive tolerance in rats using these dosage regimens [8]. However, antinociceptive tolerance differed between these dosage groups, which suggested that these dosages regimens might also differentially affect behavioral tolerance [8]. Therefore, the current study correlated behavioral and antinociceptive tolerance in rats using the same morphine dosage regimen (starting dose, follow-up dose, frequency of dosing, and duration of treatment) [8].

The current literature shows inconsistent effects of morphine on animal behavior. These inconsistencies are likely due to the use of different species/strains, routes of administration, types or formulations of morphine, age of animals, and various treatment protocols (dose, frequency, or duration of treatment) [9,10,11,12]. Locomotor activity has been widely assessed to characterize behavioral effects of morphine-treated animals. While lower morphine doses mostly left locomotor activities unaffected, higher doses produced stimulatory or biphasic effects when morphine was administered acutely [10,13,14,15,16]. Similarly, long-term morphine treatment with lower doses (1.25 to 5 mg/kg i.p.) showed no effects on locomotion, while higher doses (10 to 40 mg/kg i.p.) produced a biphasic effect with initial suppressive and subsequent increased locomotor activities [14]. Basic locomotor activity alone cannot reflect the complete behavioral side effects profile of morphine, while concomitant measurement of locomotor activities together with other behavioral parameters was shown to be better suited to model the behavioral side effects of morphine [13]. In this study, 20 mg/kg of intraperitoneal morphine increased, while 10 mg/kg of morphine decreased horizontal movements in female mice, while both doses decreased rearing and grooming activities [13]. In contrast, no dose-dependent differences were detected in male rats in response to intracerebroventricular morphine injections [10]. These discrepancies illustrate that morphine-induced behavioral changes are, among other parameters such as route of administration and species, influenced by gender. This is not surprising, since female animals are more sensitive to morphine treatment [17,18] and showed increased distance and rearing duration compared to male mice in response to morphine treatment [18]. Moreover, different environmental settings have been used for behavioral tests. Especially, changes to the illumination conditions such as brightly illuminated [18,19,20], moderately illuminated [9,13], or low illuminated environments [14] have been reported. Since rodents are more active under low illumination conditions, brightly lit open-field arenas may distract the animals, which likely alters the results compared to experiments performed under low illumination conditions. Besides, most studies only tested the animal’s acute responses 30 min after morphine administration [9,10,13], which completely disregarded the known biphasic behavioral pattern in response to morphine exposure [14,21].

Although some studies combined several behavioral activities such as distance traveled, rearing, immobility, or grooming after acute morphine treatment [9,10,13], possible connections between locomotion and other behavioral effects have not been established for repetitive long-term morphine treatment. Therefore, a significant gap of knowledge is evident regarding the relationship between antinociceptive and behavioral effects of morphine and its long-term effect on behavioral and antinociceptive tolerance. This study measured multiple behavioral effects before, during, and after long-term morphine treatment in rats. Animals were treated with two different morphine dosing regimens to establish the influence of dosing regimens on behavior. Seven behavioral parameters were measured automatically in an open-field arena, which has been rarely performed before [10]. The present study is an exploratory study to understand the behavioral tolerance profile of the same group of animals over the course of a two week treatment instead of comparing them to a different group of control animals. We aimed to generate a detailed behavioral profile of long-term morphine treatment, behavioral tolerance, and the influence of two different morphine dosage regimens, to reflect existing relationships between behavioral and antinociceptive tolerance.

## 2. Results

### 2.1. Time-Dependent Effects of a Single Dose of Morphine to Locomotor Behaviors: Hypoactivity vs. Hyperactivity

To acquire a basic understanding of how repeated morphine administration affects rat behaviors, rats were treated daily with 10 mg/kg (b.i.d.) morphine over a period of 10 days, and their activities were recorded and assessed daily at regular intervals, for a total of 180 min after administration. Daily basal levels of activity (*t* = 0 min) were similar with no significant differences over the treatment period. Therefore, no residual effects of morphine from previous administrations on the examined behavior were observed at the beginning of each daily experiment (Supplementary Figures S1 and S2). A separate control group of animals (*n* = 6) was used to assess the impact of vehicle (0.9% *w*/*v* sodium chloride solution, b.i.d.) over repeated treatments (Supplementary Figure S3). Control animals, treated similarly with the vehicle for three days, did not change locomotor behavior at the 30 min post-injection time-point. Since no differences in basal behavioral activities were observed over 14 days and no changes in the behavior of saline-treated animals over three days of consecutive treatment were detected, repeated treatment did not change the baseline behavioral activities of rats (Supplementary Figures S1–S3).

**Figure 1:**
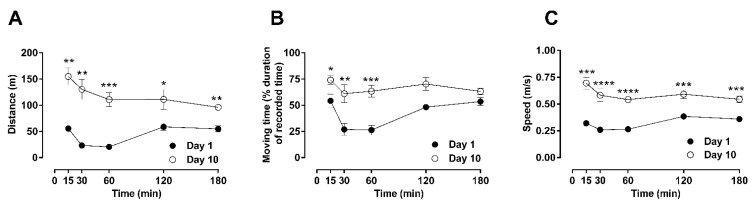
Time-resolved basic locomotor activity after repeated morphine administration (10 mg/kg, b.i.d. over 10 days). Locomotor parameters were recorded in an open-field arena after a single subcutaneous injection of morphine on day 1 or day 10 in male Sprague–Dawley rats. The motor behavior of treated animals was assessed by quantification of total distance traveled (**A**), total moving duration (**B**), or average speed (**C**), for a period of 180 min post-administration. Statistically significance (*p* < 0.05) at a specific time-point against the effects of day 1 is shown as * *p* < 0.05, ** *p* < 0.01, *** *p* < 0.001, and **** *p* < 0.0001 and was calculated using unpaired *t*-test. Values are presented as mean ± SEM (*n* = 6 animals per group). Error bars are sometimes too small to be visible. The behavioral differences between these two days are shown as the differences in their area under the curves (AUC) in Table 1, which indicates that differences were observed in the distance, moving time, and speed.

**Figure 2:**
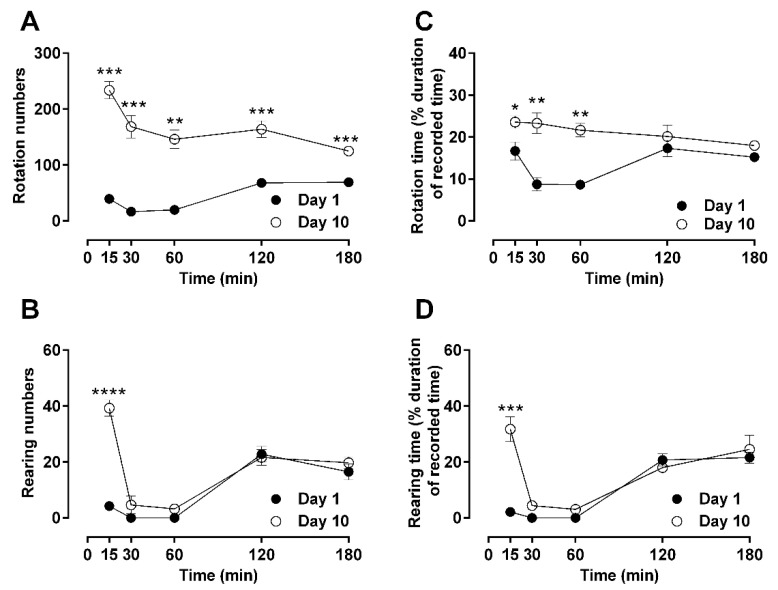
Time-resolved advanced locomotor activity (rotation and rearing) after repeated morphine administration (10 mg/kg, b.i.d. over 10 days). Open-field turning and rearing activities after a single subcutaneous injection of morphine on day 1 or day 10 were measured in male Sprague–Dawley rats. Activities of treated animals were measured as rotation numbers (**A**), rearing numbers (**B**), rotation time (**C**), and rearing time (**D**) over a period of 180 min. Statistical significance (*p* < 0.05) at a specific time-point against the effects of day 1 is shown as * *p* < 0.05, ** *p* < 0.01, *** *p* < 0.001, and **** *p* < 0.0001 and was calculated using unpaired *t*-test. Values are presented as mean ± SEM (*n* = 6 animals per group). Error bars are sometimes too small to be visible. The behavioral differences between day 1 and day 10 are shown as the differences in their area under the curves (AUC) in Table 1, which indicates that differences were observed in rotation, but not in rearing behaviors.

Behavioral scoring at every time-point was compared between days 1 and 10 using unpaired *t*-tests and is shown in Figure 1 and Figure 2 (basic locomotion, Figure 1; rearing and rotation, Figure 2). Statistical analysis was also performed using two-way ANOVA that produced similar results. For basic locomotion, at day 1 of morphine administration, suppression of locomotor activities was observed after 30 min at all parameters analyzed (one-way ANOVA; F (11, 46) = 12.43; *p* < 0.01(distance); F (11, 54) = 8.96; *p* < 0.0001 (moving time); F (11, 57) = 22.12; *p* < 0.001 (speed)), which persisted until 60 min after administration (Figure 1A–C). The repression of all examined parameters of locomotion returned to their basal levels within 180 min after administration. However, after 10 days of daily repetitive administration of morphine (day 10), general locomotion manifested as hyperactivity, as shown by significant increases in the traveled distance (one-way ANOVA; F (11, 46) = 12.43; *p* < 0.0001) and moving speed (one-way ANOVA; F (11, 57) = 22.12; *p* < 0.0001), along with non-significant differences in moving time compared to basal levels. This change in the activity profile towards hyperactivity was accompanied by a shift of its peak at 15 min after morphine administration and a faster recovery to basal levels within 180 min of treatment.

The specific locomotor behaviors of rotation and rearing were also analyzed in a similar time-resolved manner in terms of the total score (numbers; Figure 2A,C) and duration (time; Figure 2B,D). Rotational behavior was suppressed by morphine on day 1 in a similar fashion as general locomotion in terms of peak and recovery timing (one-way ANOVA; F (11, 42) = 31.56; *p* < 0.05). Similar to general locomotion, rotational behavior was also significantly increased at day 10 compared to basal levels (one-way ANOVA; F (11, 42) = 31.56; *p* < 0.01). Rearing was significantly suppressed by morphine on day 1, similarly to general locomotion (one-way ANOVA; F (11, 39) = 45.85; *p* < 0.0001). This behavior remained suppressed at day 10 without contributing to the hyperactivity usually seen in the previously recorded parameters on day 10 (Figure 2B,C).

To evaluate the overall behavioral effects of repeated morphine administration over a period of 10 days, the overall scores of Figure 1 and Figure 2 were quantified as area under the curves (AUC) and presented in Table 1. Morphine significantly stimulated locomotion after 10 days of repetitive administration compared to day 1, where it significantly suppressed locomotion, with the notable exception of rearing behavior that was persistently suppressed at day 10 (Table 1). The corresponding behavioral activities of morphine 5 mg/kg (b.i.d.) for 5 days of repeated treatment are shown in Supplementary Figures S4 and S5.

### 2.2. Dose-Dependent Effects of Repetitive Morphine Administration and Incremental Changes of Dosing on Locomotor Behavior: Hypoactivity vs. Hyperactivity

**Figure 3:**
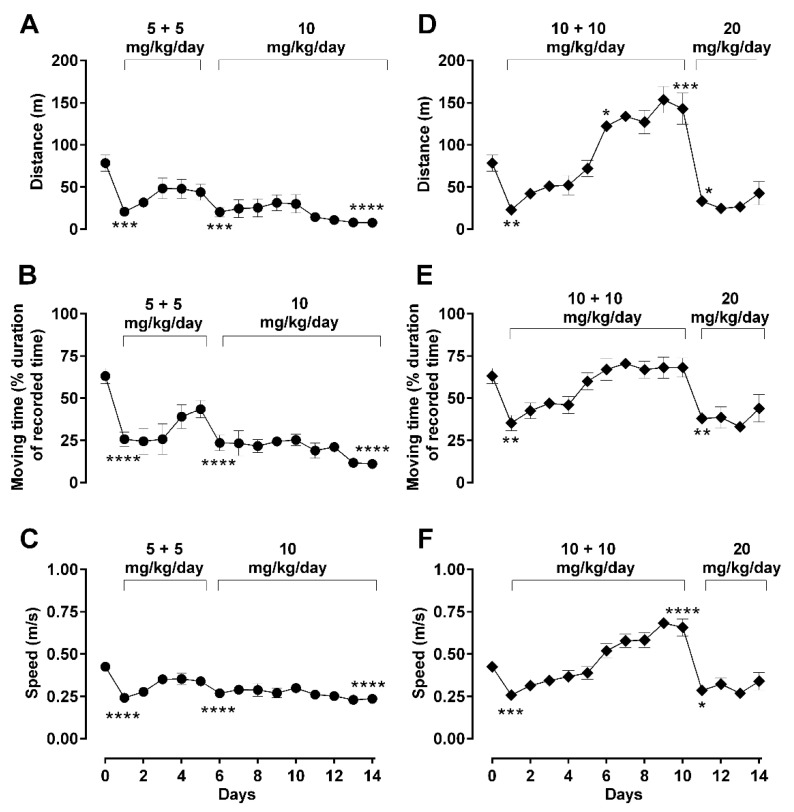
Effect of dosing regimen changes on basic locomotor activity during long-term morphine administration. Locomotor parameters were recorded daily in an open-field arena 30 min after daily subcutaneous injections of morphine in male Sprague–Dawley rats, during a 5 min recording period. Motor behavior of treated animals was assessed by quantification of distance travelled (**A**,**D**), moving duration (**B**,**E**), or average speed (**C**,**F**). Two morphine regimens were respectively used in two different groups of animals: 5 (b.i.d.) → 10 mg/kg (**A**–**C**) and 10 (b.i.d.) → 20 mg/kg (**D**–**F**) over a total period 14 days, as described in *Methods*. Values are presented as mean ± SEM (*n* = 6 animals per group). Error bars are sometimes too small to be visible. Statistically significant (*p* < 0.05) differences compared against day 0 are shown as * *p* < 0.05, ** *p* < 0.01, *** *p* < 0.001, and **** *p* < 0.0001 and were calculated using one-way ANOVA with Dunnett’s multiple comparisons test. The behavioral differences between these two dosing groups are shown as the differences in their area under the curves (AUC) in Table 2, which indicates that differences were observed in all three behavioral parameters.

To investigate the effect of morphine dosing regimens on locomotion and related behavior, animals were treated with two dosing regimens that differ in dose and duration (5 mg/kg/day b.i.d. or 10 mg/kg/day b.i.d.), followed by a subsequent change in the administration of morphine (double-dosing in single daily injections) until day 14 of treatment (Figure 3). Noticeably, no differences between basal locomotion, rotation, or rearing activities over the total treatment period of 14 days were observed, which were recorded daily (every morning) immediately before morphine injections (Supplementary Figures S1 and S2).

In the ‘low’ starting dose treatment group (5 mg/kg b.i.d. → 10 mg/kg/day; Figure 3A), moving distance was significantly reduced days 1 and 2 (one-way ANOVA; F (14, 48) = 4.26; *p* < 0.05), with a slow but steady recovery until day 5. The subsequent change in the method of administration of morphine (from twice daily 5 mg/kg to once daily 10 mg/kg) on day 6 onwards somewhat increased the suppressive effect of morphine on moving distance, returning the difference from basal back to significant levels (one-way ANOVA; F (14, 48) = 4.26; *p* < 0.001). No behavioral recovery was observed from day 6 to the end of the treatment period (day 14). The parameter of moving time (Figure 3B) and moving speed (Figure 3C) showed very similar responses as for the distance parameter. 

**Figure 4:**
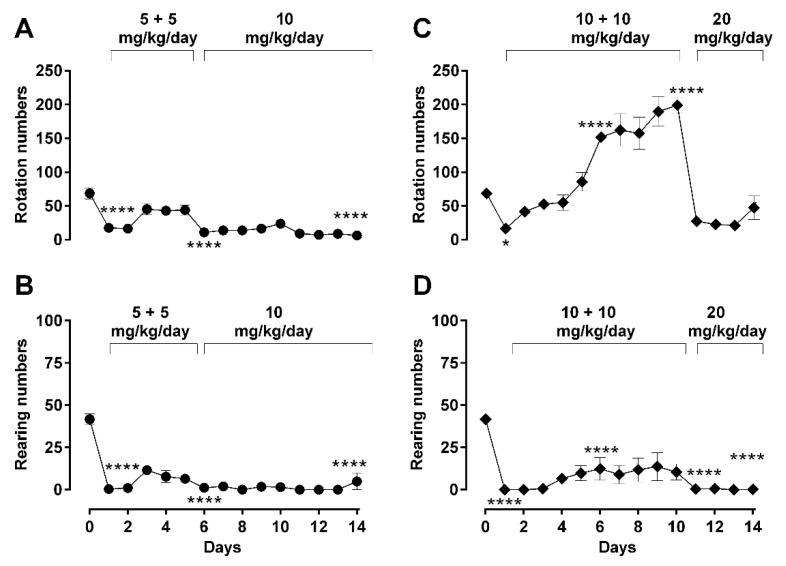
Effect of dosing regimen changes on advanced locomotor activity parameters (rearing and rotation) during long-term morphine administration. Rotation and rearing behaviors were recorded in an open-field arena after daily subcutaneous injections of morphine in male Sprague–Dawley rats, at a daily 30 min mark post-administration during a 5 min recording period. Activities of treated animals were measured as rotation numbers (**A**,**C**) and rearing numbers (**B**,**D**). Two morphine regimens were respectively used in two different groups of animals: 5 (b.i.d.) → 10 mg/kg (A, B) and 10 (b.i.d.) → 20 mg/kg (**C**,**D**) over a total period 14 days, as described in *Methods*. Values are presented as mean ± SEM (*n* = 6 animals per group). Error bars are sometimes too small to be visible. Statistically significant (*p* < 0.05) differences compared against day 0 are shown as * *p* < 0.05, and **** *p* < 0.0001 and were calculated using one-way ANOVA with Dunnett’s multiple comparisons test. The behavioral differences between these two dosing groups are shown as the differences in their area under the curves (AUC) in Table 2, which indicates that differences were observed in rotation but not in rearing behavior.

In the second group of ‘high’ morphine dosing (10 mg/kg/day b.i.d. till day 10 → 20 mg/kg/day till day 14), the same locomotive parameters were assessed, where a marked change in the overall profile was observed (Figure 3D–F). The 10 mg/kg/day b.i.d. morphine significantly reduced moving distance at day 1 compared to basal (one-way ANOVA; F (14, 47) = 19.20; *p* < 0.01); however, the recovery to basal values was not only fast and steady despite repetitive morphine administration, but also significantly increased distance traveled compared to basal values, from day 6 (one-way ANOVA; F (14, 47) = 19.20; *p* < 0.05) until it reached a hyperactivity plateau at day 10. When morphine administration changed to a single dose of 20 mg/kg/day from day 11 (Figure 3D), the suppressive effect of morphine returned to the observed levels of day 1 (one-way ANOVA; F (14, 47) = 19.20; *p* < 0.05) and remained suppressed until the end of the treatment period (day 14). This pattern of morphine-induced changes observed for traveled distance was also replicated by the other experimental parameters of general locomotion (moving time; Figure 3E and speed of movement; Figure 3F) and rotational behavior (Figure 4A,C).

For rearing behavior, a similar morphine effect during repetitive administration for both ‘low’ and ‘high’ dose groups was observed (Figure 4B,D). Morphine significantly suppressed rearing from day 1 until day 14 (one-way ANOVA; F (14, 53) = 35.57; *p* < 0.0001) without any recovery or observed increase in recorded activity, even when morphine administration was changed from twice daily to a daily single double-dose (5 mg/kg/day b.i.d. → 10 m/kg/day; Figure 4B, 10 mg/kg/day b.i.d. → 20 mg/kg/day; Figure 4D), essentially staying suppressed over the entire treatment period. 

Regimen-dependent behavioral changes were also expressed as the area under the curves (AUCs) over the whole treatment period of 14 days, and a comparison between the groups is shown in Table 2. The AUCs of behavioral parameters of the higher dosing paradigm (10 mg/kg/day b.i.d. → 20 mg/kg/day) were significantly higher than the AUCs of the lower treatment paradigm (5 mg/kg/day b.i.d. → 10 mg/kg/day), except for rearing numbers and rearing time. Thus, morphine 10 mg/kg (b.i.d.) → 20 mg/kg/day treated animals showed more locomotor and rotational behavioral changes (hyper-activity) than the morphine 5 mg/kg (b.i.d.) → 10 mg/kg/day group.

### 2.3. Relationship between Antinociceptive Tolerance and Locomotor Activities

**Figure 5:**
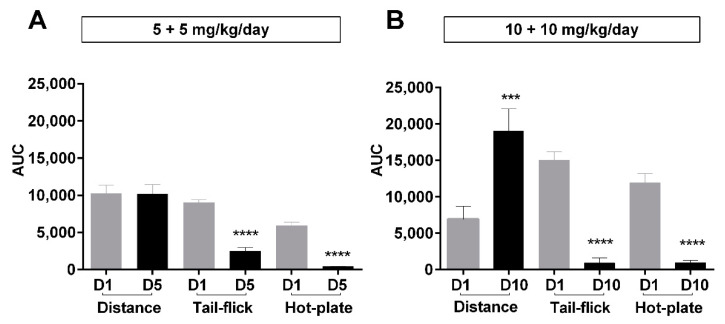
Overall behavioral and antinociceptive effects after long-term morphine administration. Area under the curves (AUC) for distance travelled and antinociception over a period of 180 min post-administration of subcutaneous morphine at different days through a treatment period. The curves used to calculate these AUC values are shown in Figure 1A and Supplementary Figures S4A and S6. Morphine was administered daily as 5 mg/kg (b.i.d.) (**A**), 10 mg/kg b.i.d. over 5 days, or (**B**) 10 mg/kg b.i.d. over 10 days. D1, D5, and D10 represent day 1, day 5, and day 10, respectively. Antinociception was assessed by tail-flick assay (TF) and hot-plate assay (HP). Values are presented as mean ± SEM (*n* = 6 animals per group). Statistically significant (*p* < 0.05) differences from day 1 for all treatment groups are shown as *** *p* < 0.001, **** *p* < 0.0001 and were calculated using unpaired *t*-tests. Error bars are present in all graphs but are sometimes too small to be visible.

To better understand the clinical significance of the biphasic behavioral effects of morphine on locomotor behavior between different dosing regimens, we aimed to compare these effects with morphine’s major pharmacological drawback, antinociceptive tolerance. Antinociception was measured using two assays (tail-flick and hot-plate), and tolerance was defined as a significant reduction in antinociceptive efficacy, whereas distance traveled was measured using open-field test over a period of 180 min after injections at day 5 or day 10, as described in *Methods*. The area under the curves (AUC) of the first treatment day (day 1), day 5 for Group A (5 mg/kg/day b.i.d.), or day 10 for Group B (10 mg/kg/day b.i.d.) were compared using unpaired *t*-tests (Figure 5). Animals in the first dose group (5 mg/kg b.i.d.) showed no difference in distance travelled between days 1 and 5, although they exhibited significant antinociceptive tolerance in both assays used (tail-flick; unpaired *t*-test; *t* (10) = 8.48; *p* < 0.0001 and hot-plate; unpaired *t*-test; *t* (10) = 9.80; *p* < 0.0001, as shown in (Figure 5A)). However, animals in the higher dose group (10 mg/kg b.i.d.) showed hyperactivity (e.g., significantly higher locomotion) at day 10 when antinociceptive tolerance first manifested, compared to day 1 (unpaired *t*-test; *t* (7) = 7.04; *p* < 0.001) (Figure 5B). These data collectively show that antinociceptive tolerance due to repetitive morphine administration is an independent effect to morphine’s effects on locomotor behavior, where the profile is largely dependent on the dosage regimen used. 

## 3. Discussion

Morphine is the gold standard for the treatment of chronic or cancer pain. Nevertheless, long-term use of morphine is severely limited by its biphasic effects on motor behavior (inhibitory or excitatory) and the manifestation of analgesic tolerance (i.e., reduced analgesic efficacy). Despite increased knowledge of the different activities of morphine, little is known about how dosing regimens affect the manifestation of motor behavioral effects. Euphoria, lethargy, or drowsiness are very common clinical side effects of morphine [22,23] and are mirrored by morphine-induced hypoactivity reported in rodents [24]. The relationship between the behavioral effects of morphine, antinociceptive tolerance, and morphine dosing has been elusive, mainly due to reported inconsistencies in experimental results, which are likely the consequence of different experimental approaches with regards to the route of administration, formulation of morphine, type of animals used, as well as treatment protocols (dose, frequency, or duration of treatment) [9,10,12,25]. All these experimental variables are likely to influence the effect of morphine on motor behavior and therefore highlight the need to examine the relationship between morphine’s behavioral effects and dosing in a unified model. Understanding how morphine dosing contributes to behavior is crucial for future clinical strategies to reduce morphine’s side effects.

We assessed hypoactivity by monitoring the major parameters of locomotor activity (distance, speed, moving time, rotational behavior, and rearing), as surrogate markers for morphine-induced motor side effects. Locomotor activity was previously used to assess behavioral side effects after acute or chronic treatment with morphine [12,13,14]. However, basic locomotion alone cannot represent all facets of drug-induced behavioral changes—combining it with additional behavioral parameters is a much more promising approach [13].

This study confirmed that subcutaneous administration of morphine produces locomotor suppression in rats after acute administration, which is in agreement with previous studies [10,13,14,26]. We also showed that repetitive morphine administration (twice a day) resulted in locomotor tolerance after 5 days, the extent of which depended on the dose administered (partial tolerance at 5 mg/kg b.i.d. and full tolerance at 10 mg/kg b.i.d.). The tolerance profile for the behavioral effect of morphine seemed to manifest in parallel to antinociceptive tolerance, as we recently described [8]. Similar to the basal antinociceptive effects reported previously [8], basal locomotor activities (locomotor, rotation, and rearing activities) were also unaffected by repeated morphine dosing over two weeks, which supports a previous report [17]. Therefore, morphine showed no residual effects on behavior or antinociception after repeated dosing over two weeks. Noticeably, no behavioral changes in control rats were observed in comparison to the effects of day 1, which indicated that the 0.9% *w*/*v* sodium chloride solution or repeated measurements did not affect the development of behavioral tolerance. However, future follow-up studies should include longer periods of vehicle treatment, similar to morphine treatment regimens to provide more evidence for this observation.

The biphasic motor effect of morphine has been known for quite a while but has been mainly described in acute and short-term morphine administration protocols [9,12,13,14,26,27,28]. The present study described the manifestation profile of behavioral excitatory state (i.e., rate of increase and timing of expression) in detail, which is vital to understand the underlying mechanisms. This study also described the effect of the morphine dosing regimen for the expression of morphine-induced hyperactivity. When the morphine dosing regimen changes after the occurrence of hyperactivity, such as 10 mg/kg (b.i.d.) → 20 mg/kg/day (once daily), the strong suppressive effect of morphine returns to the pro-hyperactivity levels and results in morphine-induced hypoactivity manifestation (Figure 3). Our data also show that the morphine-induced excitatory effect results from morphine-induced tolerance on motor behavior, which can be reversed by an increased dose, similarly to how antinociceptive tolerance can be reversed by a dose increase [8].

Activation of the MOP receptor by an agonist (e.g., morphine) increases dopamine levels in the brain [29,30]. Morphine-induced hyperactivity can be reduced by blocking dopaminergic receptors, but these are not specific to the dopaminergic system [12,31]. The higher AUC of behavioral activities of animals treated with morphine over a few days clearly indicates that the locomotion-related behavioral effects changed over this time using a specific dose. In our study, the effects of morphine were dependent on the dose administered and the dosage regimen, as the AUCs of locomotor activities were statistically lower (over the entire 14 days) with lower doses compared to the higher dose group.

Opioid-induced turning, circling, or rotation is mainly mediated by the dopaminergic system [32,33], and circling animal models have been used to assess anti-Parkinson’s disease drugs [34]. The rotating or turning behavior in this study was suppressed in line with a suppression of general locomotion after acute treatment of morphine with a similar time kinetic, which replicates previous studies [35,36]. We also showed that the rotational behavior is subjected to morphine-induced tolerance, similarly to locomotion (Figure 2), suggesting a strong link of this behavior to the opioidergic system.

Rearing activity is an exploratory behavior of rodents related to information gathering or cognitive behavior [37]. Little or no rearing in the open field may indicate motor impairment [38]. Therefore, unsurprisingly, changes in rearing behavior are also influenced by benzodiazepine treatment [39], which suggests that other neuronal systems might be more important for this behavior. Rearing is related to gamma-aminobutyric (GABA) neurotransmission controlled by the GABA_A_ receptor in the hippocampus [40]. Locomotion and rearing are positively correlated and are very reliable factors for exploratory behavior in untreated animals [9,41]. Here, we showed that morphine reduces rearing activities due to repetitive treatment with both low and high doses of morphine, in line with previous studies [42]. However, the morphine-induced suppression of rearing was not associated with tolerance, irrespective of the morphine dose used, the changes in dosing regimen, and the length of treatment (Figure 2). The different effects on rearing behavior compared to the rest of the motor behaviors tested (free moving and rotation) could indicate that additional non-opioidergic systems that affect brain areas involved in motor control of this behavior alleviate or delay the manifestation of tolerance in rearing.

In summary, our results illustrate that a lower morphine dose reduced motor behavior, which was subject to behavioral tolerance after repetitive administration but did not lead to subsequent behavioral hyperexcitation. In contrast, animals treated with a higher morphine dose developed acute motor-suppressive behavior that quickly desensitized to basal levels and progressed to an excitatory phase after 10 days, which was parallel to the development of antinociceptive tolerance [8]. Therefore, morphine dosing plays a crucial role in the manifestation of motor behavioral tolerance that follows a similar pattern to antinociceptive tolerance. The kinetics of morphine-induced suppression of motor behavior and subsequent behavioral tolerance were similar to those of antinociception and antinociceptive tolerance, as reported previously [8]. In contrast, rearing showed a distinctive resistance to tolerance and dosing changes. Our results suggest that morphine dosing determines the expression profile of behavioral effects by morphine and that antinociceptive tolerance is linked to the morphine-induced hyper-excitatory phase of behavior.

## 4. Materials and Methods

### 4.1. Animal Maintenance and Care

Eighteen male Sprague–Dawley (SD) rats (234.0 ± 6.1 g, 8 weeks) were housed as three littermates per cage at 22 °C with 50–60% humidity under an automated 12-h day/night cycle (lights on at 7:00 a.m.) with free access to food and water. All procedures were approved by the University of Tasmania Animal Ethics Committee (A0013864) and were conducted according to The Australian Code for the Care and Use of Animals for Scientific Purposes and in compliance with the ARRIVE guidelines [43,44]. Animals were handled for 5–6 days before starting the experiments and acclimatized to the test environment for 2 h in home cases prior to daily experiments.

### 4.2. Treatment Protocol

Body weight was recorded daily immediately before experiments. Animals were divided into three subgroups using a completely randomized design as previously described [45]. Two sub-groups of animals received different morphine dosing. Group A (*n* = 6): morphine sulfate 5 mg/kg (twice daily) for 5 days, followed by a single dose (once daily) of 10 mg/kg from day 6 to 14. Group B (*n* = 6): morphine sulfate 10 mg/kg (twice daily) for 10 days, followed by a single dose (once daily) of 20 mg/kg from day 11 to 14, as described in a previous study [8]. Commercially available 30 mg/mL morphine sulfate solution (Hameln Pharmaceuticals GmbH, Hamelin, Germany) was administered by daily subcutaneous injections between the left thigh and the spinal cord for group B. For group A, morphine sulfate was diluted to 15 mg/mL with sterile 0.9% *w*/*v* sodium chloride solution immediately before injections, as we described previously in another study [15]. The third sub-group, group C (*n* = 6), was a control group of animals were treated similarly with sterile 0.9% *w*/*v* sodium chloride solution in water (b.i.d.) for 3 days. Injection volumes, morphine doses, dilution, and timing of injections were selected based on our previous results [8]. The illumination intensity of the laboratory was reduced prior to and during experiments to minimize discomfort to the animals. At the end of each study, animals were anesthetized with 5% (*w*/*v*) isoflurane in oxygen at a flow rate of 1 L/min before decapitation.

### 4.3. Locomotor Activity Measurements

**Figure 6:**
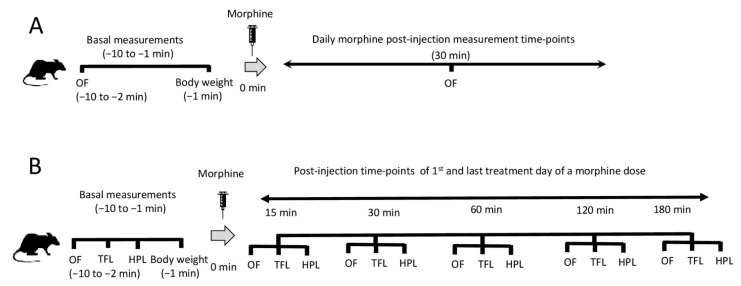
Schematic representation of the morphine treatment as well as behavioral and antinociception assessments used in the study. Animals were treated daily for baseline behavior (pre-) and 30 min post-administration of morphine (**A**), or pre- and post- (15, 30, 60, 120 and 180 min) administration of morphine on the first and the last treatment day of a morphine dose (**B**). The main assessment of motor behavior was calculated using data from open-field tests (OF). Antinociception was followed in order to validate the effect of morphine on the animals and be able to relate morphine’s antinociceptive effect with the produced motor behaviors after repeated drug administrations. Antinociception assessment was performed using tail-flick (TFL) and hot-plate (HPL) tests.

Behavior was tested in an open-field arena in an automated Multi-Conditioning System (MCS) (TSE GmbH, Homburg, Germany) pre- and post- (15, 30, 60, 120 and 180 min) administration of morphine over 5 min on the first and the last treatment day of the same dose, since a 5 min observation period is widely used [9,13,15]. On all other treatment days, the rats were tested only for baseline behavior (pre-) and 30 min post-administration of morphine, representing the time-point of morphine-induced maximal behavioral suppression on day 1. Similarly, open-field behaviors of control-group animals were measured after 30 min post-injection time-point for three consecutive days. Behavioral testing included seven different parameters (moving time, total distance traveled, speed, rotation numbers, rearing numbers, rotation time, and rearing time). Speed (m/s) was calculated as distance traveled (m) divided by the corresponding moving time (s). Rotation numbers were calculated as the sum of clockwise and counterclockwise rotations. MCS included an internal noise/light/temperature insulation system and a 3D infrared beam frame that provided fast (100 Hz) and accurate animal movement (TSE ActiMot) combined with high-resolution video monitoring. The open-field arena was thoroughly cleaned and dried between each animal. A background noise (20 dB) was used to cancel out any unexpected laboratory sounds during experiments. The area under the curves (AUC) was calculated by the trapezoid method using GraphPad Prism V6 software (GraphPad Software Inc., La Jolla, CA, USA). Morphine treatment and behavioral measurements at different time-points are represented in a schematic diagram (Figure 6).

### 4.4. Assessment of Antinociception

Nociceptive thresholds were measured by tail-flick and hot-plate assay using equipment purchased from Ugo Basile (Comerio, Italy. Animals were tested randomly to avoid any bias effects due to multiple repeated measurements. The maximum exposure to the nociceptive thermal stimulus was 15 s for the tail-flick (basal latency: 4.3 ± 0.2 s) and 30 s for the hot-plate assay (basal latency: 5.7 ± 0.3 s). The infrared intensity of the tail-flick photocell was set at 30, whereas the plate temperature of the hot-plate was set at 54 ± 0.5 °C. The experimental settings for antinociceptive measurements used in this study were previously described [8,46,47,48]. Every rat was tested immediately prior to morphine administration as well as 15, 30, 60, and 120 min post-administration using both assays only on the first and the last treatment day (Figure 6). Measurements were conducted in a blinded manner, and the mean of three independent measurements for each time-point with a 1 min interval between measurements was recorded to minimize the operator’s handling effects. The maximum possible effect (MPE) was defined as MPE % = 100 × [(test latency–basal latency)/(cut-off time–basal latency)] as previously described [49]. The area under the curves (AUC) was calculated by the trapezoid method using GraphPad Prism V6 software (GraphPad Software Inc., La Jolla, CA, USA).

### 4.5. Statistical Analysis

The area under the curves (AUC) was calculated by the trapezoid method using GraphPad Prism V6 software (GraphPad Software Inc., La Jolla, CA, USA). Data are expressed as mean ± SEM and analyzed by one-way ANOVA with Dunnett’s multiple comparisons test (for comparisons between three or more animal groups) or unpaired *t*-test (for comparisons between two groups of animals). Multiple comparisons (Dunnett’s test) were employed when F achieved *p* < 0.05, and there was no significant variance in the homogeneity. A ‘*p*’ value less than 0.05 was considered statistically significant.

## Figures and Tables

**Table 1:** Comparison of motor behaviors from day 1 after repeated morphine administration. Total scoring of motor behavior was recorded over 180 min after subcutaneous administration of morphine 10 mg/kg (b.i.d.) over 10 days of repeated treatment. Parameters (presented as the area under curve; AUC) were calculated from the behavioral curves in Figure 1 and Figure 2. Statistically significant differences between AUCs of each behavior were assessed using an unpaired *t*-test.

Behavioral Parameter	AUC Units	Day 1 (Dose Group B)	Day 10 (Dose Group B)	Significance (*p*-Value)
Distance	meter × days	8958 ± 995	19,492 ± 1562	<0.001
Moving time	% of recorded time × days	8349 ± 643	11,715 ± 643	<0.01
Speed	(meter/sec) × days	60.22 ± 1.25	99.51 ± 3.34	<0.0001
Rotation numbers	incidences × days	8374 ± 550	25,423 ± 1761	<0.0001
Rotation time	% of recorded time × days	2464 ± 240	3704 ± 199	<0.01
Rearing numbers	incidences × days	3018 ± 308	3717 ± 206	0.096
Rearing time	% of recorded time × days	2116 ± 171	2803 ± 458	0.2269

**Table 2:** Overall comparison of motor behaviors between different dosing groups. Total scoring of recorded motor behaviors (presented as area under curve; AUC) were calculated from the behavioral curves in Figure 3 and Figure 4. Statistically significant differences between AUCs of each behavior were assessed using an unpaired *t*-test.

Behavioral Parameter	AUC Units	Dose Group A	Dose Group B	Significance (*p*-Value)
Distance	meter × days	369.6 ± 71.6	982.3 ± 134.3	<0.01
Moving time	% of recorded time × days	305.5 ± 32.6	700.2 ± 51.4	<0.001
Speed	(meter/sec) × days	3.80 ± 0.23	5.69 ± 0.42	<0.01
Rotation numbers	incidences × days	255.8 ± 48.4	1200 ± 124.2	<0.001
Rotation time	% of recorded time × days	103.1 ± 12.6	233.4 ± 22.2	<0.001
Rearing numbers	incidences × days	48.0 ± 15.5	97.4 ± 30.1	0.174
Rearing time	% of recorded time × days	19.4 ± 2.86	40.6 ± 9.9	0.130

## Data Availability

Not applicable.

## Supplementary Materials

The following are available online. Figure S1: Basal basic locomotor activities of rats. Figure S2: Basal advanced locomotor activities (rotation and rearing) of rats. Figure S3. Locomotor activities of control animals after repeated treatment. Figure S4: Time-resolved basic locomotor activities after repeated morphine treatment. Figure S5: Time-resolved advanced locomotor activities (rotation and rearing) after repeated morphine administration. Figure S6: Antinociceptive effects of daily morphine treated rats.
