# Supplementary material for: Profiling the Effects of Repetitive Morphine Administration on Motor Behavior in Rats
Source: Molecules. 2021 Jul 19;26(14):4355. doi: 10.3390/molecules26144355 (PMC8308092; doi:10.3390/molecules26144355)
Supplement: Supplementary file 1 [file molecules-26-04355-s001.zip › molecules-1284169-supplementary.pdf]

Supplementary materials for

Profiling the effects of repetitive morphine administration on motor behavior in rats

Alok K. Paul [1]*, Nuri Gueven [1] and Nikolas Dietis [2]*

[1] School of Pharmacy and Pharmacology, University of Tasmania, Hobart, TAS 7001, Australia; alokkpaul@gmail.com (A.K.P.); nuri.guven@utas.edu.au (N.G.)

[2] Medical School, University of Cyprus, Nicosia, Cyprus; dietis.nikolas@ucy.ac.cy (N.D.)

*Correspondence: alokkpaul@gmail.com (A.K.P.); dietis.nikolas@ucy.ac.cy (N.D.)

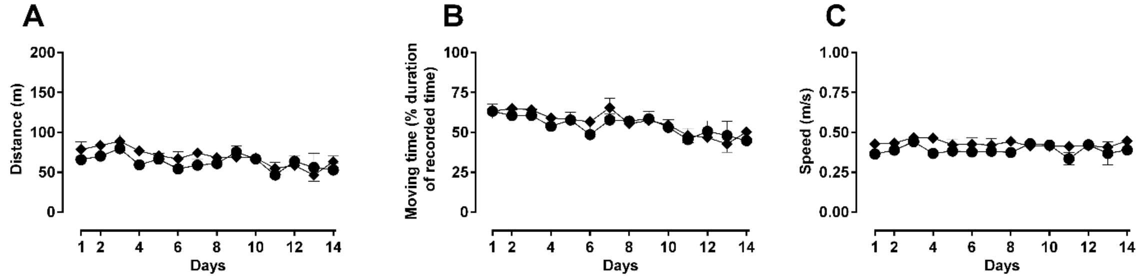

Supplementary Figure S1. Basal basic locomotor activities of rats. Open-field locomotor activities at pre-administration (basal) in male Sprague Dawley rats over a period of 14 days. Activities of animals were measured as distance traveled **(A),** moving time **(B)**, or speed of movement **(C)**. Values are presented as Mean ± SEM (n = 6 animals per group). Error bars are sometimes too small to be visible. No statistically significant differences were observed against day 1 using one-way ANOVA with Dunnett's multiple comparisons tests.

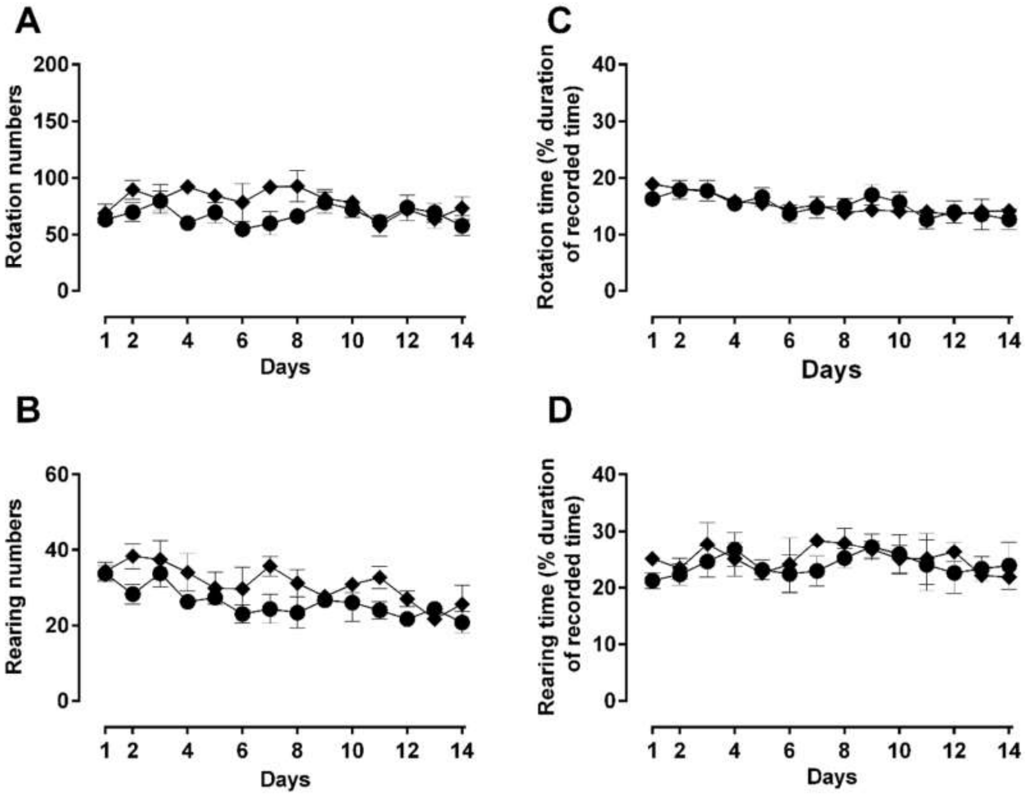

Supplementary Figure S2. Basal advanced locomotor activities (rotation and rearing) of rats. Open-field rotation and rearing activities at pre-administration (basal) in male Sprague Dawley rats over a period of 14 days. Activities of animals were measured as rotation no **(A)**, rearing no **(B)**, rotation time **(C)** and rearing time **(D)**. Values are presented as Mean ± SEM (n = 6 animals per group). Error bars are sometimes too small to be visible. No statistically significant differences were observed against day 1 using one-way ANOVA with Dunnett's multiple comparisons tests.

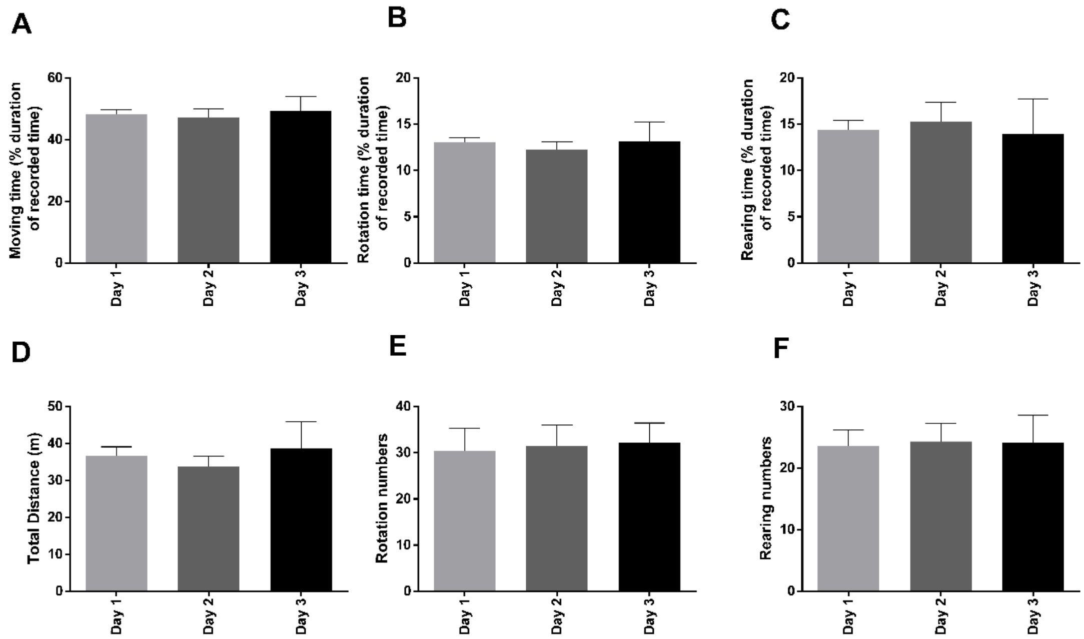

Supplementary Figure S3. Locomotor activities of control animals after repeated treatment. Locomotor parameters were recorded in an open-field arena daily over consecutive 3 days after subcutaneous injections of 0.9% w/v saline solution (b.i.d.) in male Sprague Dawley rats, at a daily 30 min mark post-administration during a 5 min recording period. The motor behavior of treated animals was assessed by quantification of moving duration **(A)**, rotation duration **(B)**, rearing duration **(C)**, distance travelled **(D)**, rotation numbers **(E)**, or rearing numbers **(F)**. Values are presented as mean ± SEM (n = 6 animals per group). Error bars are sometimes too small to be visible. Statistically significant ($p<0.05$) differences compared against day 1 were calculated using one-way ANOVA with Dunnett's multiple comparisons test, but no significant changes were recorded.

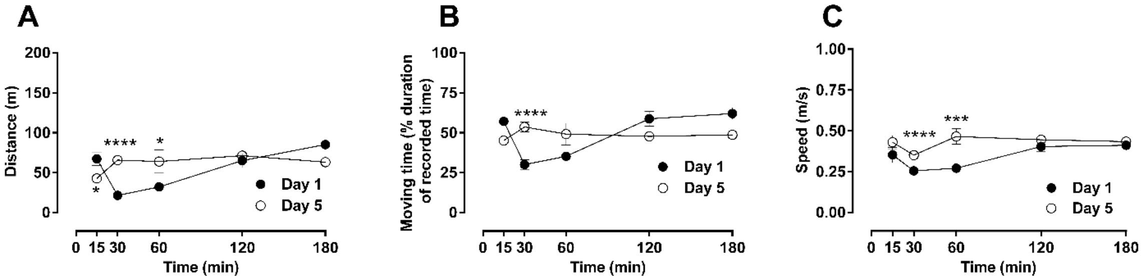

Supplementary Figure S4. Time-resolved basic locomotor activities after repeated morphine treatment (5 mg/kg, b.i.d. over 5 days). Open-field locomotor activities after a single subcutaneous injection of morphine on day 1 or day 5 were measured in male Sprague Dawley rats. Activities of treated animals were measured as distance traveled (A), moving time (B), or speed of movement (C) over a period of 180 min. Statistically significance ($p<0.05$) at a specific time-point against the effects of day 1 is shown as *$P<0.05$, **$P<0.01$, ***$P<0.001$ and ****$P<0.0001$ and was calculated using unpaired t-test. Values are presented as mean ± SEM (n = 6 animals per group). Error bars are sometimes too small to be visible.

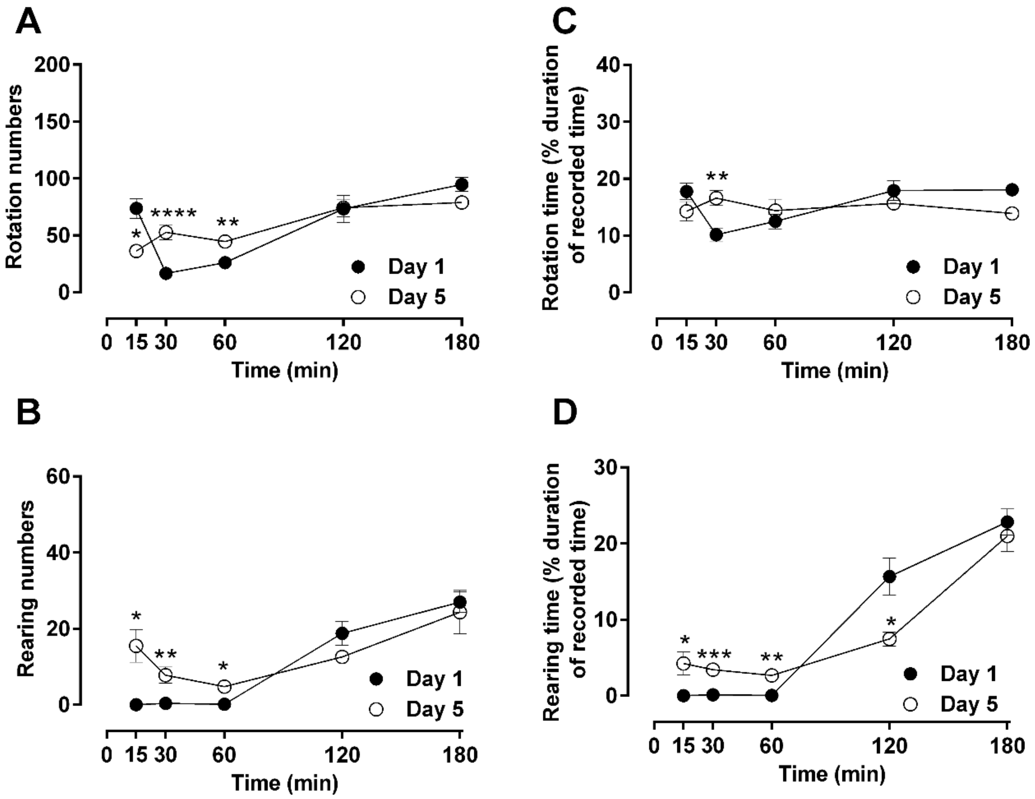

Supplementary Figure S5. Time-resolved advanced locomotor activities (rotation and rearing) after repeated morphine administration (5 mg/kg, b.i.d. over 5 days). Open-field turning and rearing activities after a single subcutaneous injection of morphine on day 1 or day 5 were measured in male Sprague Dawley rats. Activities of treated animals were measured as rotation no **(A)**, rearing no **(B)**, rotation time **(C)** and rearing time **(D)** over a period of 180 min. Statistically significance ($p<0.05$) at specific time-point against the effects of day 1 is shown as $^*P<0.05$, $^{**}P<0.01$, $^{***}P<0.001$ and $^{****}P<0.0001$ and was calculated using unpaired t-test. Values are presented as mean ± SEM (n = 6 animals per group). Error bars are sometimes too small to be visible.

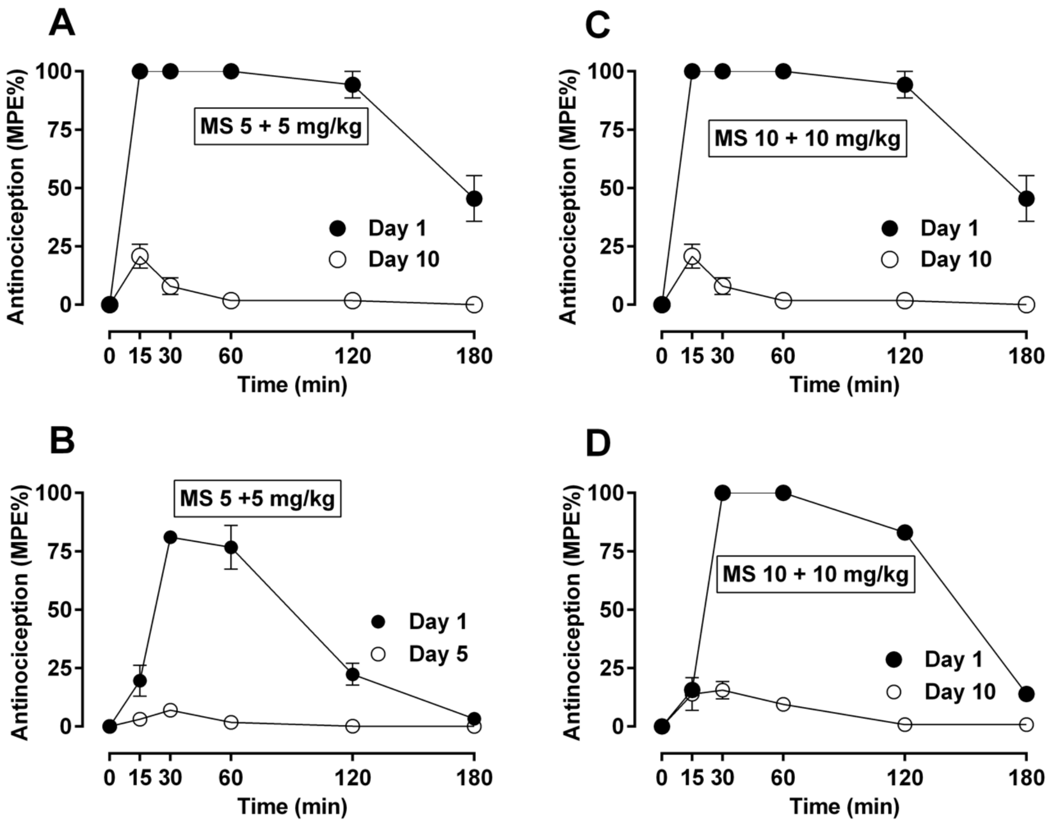

Supplementary Figure S6. Antinociceptive effects of daily morphine treated rats. Antinociceptive effects of 5 mg/kg (b.i.d.) and 10 mg/kg (b.i.d.) morphine treated Sprague Dawley rats (n = 6 per group) over the course of 5 to 10 days (groups A and B). The antinociceptive effects were measured using tail-flick (A, C) and hot-plate (B, D) assays. The area under the curves were shown in Fig. 5. Values are presented as mean ± SEM (n = 6 animals per group). Error bars are sometimes too small to be visible.
